# Geographical adaptability for optimizing the recommendation of soybean cultivars in the Brazilian Cerrado

**DOI:** 10.1038/s41598-024-63809-y

**Published:** 2024-06-06

**Authors:** Marcos Corbellini, Daniel Vicente Bobek, José Francisco Ferraz de Toledo, Lenio Urzeda Ferreira, Dthenifer Cordeiro Santana, Thiago Alexandre Santana Gilio, Larissa Pereira Ribeiro Teodoro, Paulo Eduardo Teodoro, Flavio Dessaune Tardin

**Affiliations:** 1 Syngenta Seeds, Lucas do Rio Verde, MT 78455-000 Brazil; 2 The Context Network, Sorriso, MT 78603-237 Brazil; 3 Federal University of Mato Grosso do Sul (UFMS), Chapadão do Sul, MS 79560-000 Brazil; 4 Universidade Federal de Mato Grosso (UFMT, Sinop), Sinop, MT 78550-000 Brazil; 5 Embrapa Milho e Sorgo, Sete Lagoas, MG 35701-970 Brazil

**Keywords:** Plant sciences, Environmental sciences

## Abstract

Yield multi-location trials associated to geostatistical techniques with environmental covariables can provide a better understanding of G x E interactions and, consequently, adaptation limits of soybean cultivars. Thus, the main objective of this study is understanding the environmental covariables effects on soybean adaptation, as well as predicting the adaptation of soybean under environmental variations and then recommend each soybean cultivar to favorable environments aiming maximize the average yield. The trials were carried out in randomized block design (RBD) with three replicates over three years, in 28 locations. Thirty-two genotypes (commercial and pre-commercial) representing different maturity groups (7.5–8.5) were evaluated in each trial were covering the Edaphoclimatic Region (REC) 401, 402 and 403. The covariables adopted as environmental descriptors were accumulated rainfall, minimum temperature, mean temperature, maximum temperature, photoperiod, relative humidity, soil clay content, soil water avaibility and altitude. After fitting means through Mixed Linear Model, the Regression-Kriging procedure was applied to spacialize the grain yield using environmental covariables as predictors. The covariables explained 32.54% of the GxE interaction, being the soil water avaibility the most important to the adaptation of soybean cultivars, contributing with 7.80%. Yield maps of each cultivar were obtained and, hence, the yield maximization map based on cultivar recommendation was elaborated.

## Introduction

In recent years, meeting the rapid growth of the world's population in terms of food security and jointly overcoming the limited expansion of areas for agricultural production has been a major challenge^1^. In an effort to overcome these challenges, several technological advances in crop management and plant breeding have been implemented in Brazil. The latter includes the adaptation of new cultivars to low latitudes for growing soybean crop, one of the world's most important agricultural commodities^2^.

The environment has a major influence on the phenotypic behavior of soybeans, especially through edaphoclimatic factors such as water availability, temperature, and photoperiod. One of the main objectives of plant breeding is to develop new cultivars that are highly productive and broadly adapted to different growing environments^3^. The use of environmental information in network trial analysis can provide a better interpretation of the genotype-environment interaction (GxA), making it possible to predict the behavior of cultivars in non-sampled environments by estimating the reaction norms of each genotype^1,4^. Thus, the reaction norm (which comes from the German, reaktionsnorm = response pattern curve) is the responsiveness of the genotype to some environmental variation^5^.

Overall, the soybean crop, like other plant species, has a certain thermal demand for regular development. However, extreme temperatures, whether high or low, can result in highly unfavorable heat stress for the crop. Excessively high temperatures cause excess evapotranspiration with consequent dehydration and reduced photosynthetic efficiency^6–8^. Similarly, low-temperature stress limits plant growth and development processes by reducing metabolism. This type of stress also affects other physiological processes, such as membrane fluidity, water and nutrient uptake, protein and nucleic acid conformation^9–11^ when evaluating the effect of heat stress on soybeans by raising the atmospheric temperature, found a reduction in the number of pods and seeds per plant. Similar studies carried out by^12^ in a greenhouse to determine the effect of climatic variables, showed that high temperatures contributed to the loss of biomass and grain yield.

Multi-environment trials associated with geostatistical techniques, with the support of environmental variables, can help to understand the G × A interaction and the adaptive limits of soybean cultivars, by modeling reaction norms across different environments. The aim is to simulate the behavior of cultivars in environments not included in the experimental network^1^. In Brazil, the recommendation of soybean cultivars is usually geared towards states and municipalities, using, directly or indirectly, political boundaries as indicators for structuring environmental variation. This brings simplicity to the recommendation, but can result in low technical assertiveness in defining the adaptive region of each cultivar. In view of this difficulty, recommendations based on the spatialization of yields, supported by the use of covariates, result in greater precision for recommending cultivars, since environmental variation is indicated by spatial trends in yields and by variations in the values of each covariate. Assuming that there is a geographical position where a yield of a cultivar is maximized, the point of greatest adaptation, as it moves away from this point, grain yield tends to decrease, indicating that the cultivar is moving away from the adaptive center. Based on this behavior, recommendation maps can be drawn up.

Among the various techniques that can be used for the spatial modeling of reaction norms, Regression-Kriging stands out, as it consists of the spatialization of a given variable using the Kriging procedure and multiple regression of predictors. In this context, the aim of this study was to: (i) understand the adaptation of soya cultivars in the state of Mato Grosso, by spatializing productivity, in order to know the adaptive geographical limits of soya cultivars; (ii) estimate the effects of geographical positioning and environmental variables on the adaptation of soya cultivars in this region; (iii) draw up maps for recommending cultivars, with a view to maximizing productivity.

## Results

### Deviance analysis and genetic selection of the best cultivars

The G significance indicates that there are differences between the yields of the cultivars at genetic level across all the environments sampled. On the other hand, significant differences in the GxE interaction (p < 0.05) show the different behavior of the cultivars across environments (Table 1).

**Table 1:** Results of the analysis of variance, variance components, and coefficients of determination (R^2^) referring to the joint analysis for grain yield of 32 soybean cultivars evaluated over three subsequent harvests (2019/2020 to 2021/2022) in 89 environments in the State of Mato Grosso.

Factor^1^	Deviance	LRT (χ^2^)	Variance	Standard deviation	R^2^
Genotype (G)	210,054	557.12*	53,578.73	231.47	16.29%
Environment (E)	210,504	1006.78**	200,288.54	447.54	31.50%
G × E	212,400	2903.3*	55,650.96	235.90	16.61%
Block	210,911	1414.5*	44,831.90	211.74	14.91%
Residual	–	–	86,388.07	293.92	20.69%
Model	224,486	–	–	–	–

^1^G, G × E correspond to genetic and genotypes by environment interaction effects; *and**significant values by the chi-square test at 5% or 1% probability.

There is considerable proximity between the average yields, which is due to the fact that the set of cultivars is summarized as the eight most productive. The most productive cultivar was G-19 (Table 2).

**Table 2:** Average grain yield ($\widehat{u}$+$\widehat{g}$), in Kg ha^−1^, and genetic effects for the eight best soybean cultivars over three subsequent crops (2019/2020 to 2021/2022) grown in 89 environments in Mato Grosso.

Code^1^	RM	$\widehat{g}$	$\widehat{u}$+$\widehat{g}$	Winning %^2^
G-19	8.2	363.05	4213.80	28.09%
G-06	7.6	275.62	4126.37	17.98%
G-11	7.8	316.84	4167.59	11.24%
G-17	8.1	267.37	4118.12	8.99%
G-20	8.0	199.80	4050.55	7.87%
G-22	8.3	240.29	4091.04	12.36%
G-25	8.4	248.04	4098.79	7.87%
G-07	7.6	122.58	3973.33	5.62%
Intercept	3850.75			

^1^Reference codes of the cultivars evaluated; ^2^Proportion of locations in which a cultivar was the most productive.

### Effects of environmental covariates on the adaptation of cultivars

Looking at the average coefficients (Table 3), it can be seen that all the environmental variables were significant (p < 0.05) regarding grain yield. However, there are variations in the magnitude and significance of the environmental covariate effects at cultivar level, with some variables being more relevant for specific cultivars. These coefficients directly represent the reaction norms of each cultivar associated with the different environmental factors. Mathematically, they represent the weightings of the effects of each covariate on yield. Thus, by multiplying each coefficient by the value of the respective covariate in a given spatial position, the predicted reaction norm is obtained.

**Table 3:** Linear coefficients, overall and detailed by cultivar, for the environmental variables used in the multiple linear regression.

Variable	Cultivars								Overall
	G-06	G-07	G-11	G-17	G-19	G-20	G-22	G-25	
ALT	1.4614*	0.4009*	0.4088*	0.2589^ns^	0.9259*	− 0.2470^ns^	0.3112^ns^	0.5026*	0.4658*
SWA	3.8238*	2.7094^ns^	3.7397^ns^	6.6260*	6.2002*	9.0234*	4.7691*	3.7899^ns^	4.7510*
PHP	4.4811*	1.0843^ns^	2.7859*	1.6255^ns^	1.2693^ns^	2.5223*	5.2680*	2.0357*	2.2897*
Pa	1.0334^ns^	2.7330*	0.5615^ns^	1.2453^ns^	1.3040*	3.4971*	2.1356*	2.3715*	1.6121*
CC	1.9418^ns^	3.1945*	2.5289*	2.3124*	2.5200*	1.9758^ns^	1.6528^ns^	2.5436*	2.2911*
Tmax	− 4.0196*	− 4.0643*	− 1.0542^ns^	− 1.4128*	− 0.6406^ns^	4.1951*	− 1.8829*	− 3.6742*	− 2.1475*
Tm	1.0997^ns^	− 1.2756^ns^	1.6585*	7.4065*	3.2933*	3.0928*	0.6009^ns^	1.0019^ns^	1.7906*
Tmin	1.6937*	0.8017^ns^	1.1386*	2.5862*	− 2.2580*	2.0745*	1.0507^ns^	0.6129^ns^	1.3651*
RH	0.2484^ns^	1.7926*	1.9524*	2.9199*	0.5207^ ns^	1.9836*	1.2339^ns^	3.5303*	1.3559*

*ALT* altitude, *SWA* soil water availability, *PHP* photoperiod, *Pa* accumulated precipitation, *CC* clay content in the soil, *Tmax* maximum air temperature, *Tm* mean air temperature, *Tmin* minimum air temperature, *UR* relative humidity. Measurement of RH based on two meters above ground level; *and**significant values at 5% or 1% probability; and ^ns^non-significant values.

Cultivar G-07 had the lowest mean yield. However, even though cultivar G-19 was the most productive on average in all the locations sampled, there is a considerable proportion of locations where this behavior is not repeated. Likewise, despite having the second lowest mean yield, G-25 had the highest individual yield in specific environments. Therefore, it is necessary to understand the adaptive limitations of each cultivar in order to make appropriate recommendations for its use.

Table 4 shows differences between the average coefficients of determination (R^2^) related to each covariate, which correspond to the proportion of variation observed in grain yield considering all the cultivars. On average, SWA had the highest ratio (7.80%), while RH had the lowest ratio with productivity (4.64%). There is also a considerable difference in the R^2^ values related to each covariate between cultivars. This is due to the different responses that each cultivar shows to environmental variation. Therefore, differences are also observed in the R^2^acum, which corresponds to the total variation explained by the covariates per cultivar. In this case, cultivar G-22 had the lowest value (18.49%), while G-20 achieved the highest value (42.73%).

**Table 4:** Coefficient of determination (R^2^) of the environmental variables (predictors) on the grain yield of soybean cultivars.

Covariable^1^	G-06	G-07	G-11	G-17	G-19	G-20	G-22	G-25	R^2^_mean_
ALT	10.86%	5.92%	5.63%	–	7.21%	–	–	6.28%	7.18%
SWA	6.55%	–	–	8.87%	7.91%	10.10%	5.58%	–	7.80%
PHP	5.12%	–	4.19%	–	–	4.01%	5.77%	3.23%	4.46%
Pa	–	4.13%	–	–	3.99%	6.49%	5.15%	5.80%	5.11%
CC	–	8.65%	6.54%	5.15%	5.27%	–	–	6.29%	6.38%
Tmax	8.17%	7.90%	–	2.15%	–	6.14%	2.39%	7.51%	5.59%
Tm	–	–	3.19%	9.73%	4.44%	4.20%	–	–	5.39%
Tmin	4.14%	–	4.21%	6.18%	5.33%	7.43%	–	–	5.46%
RH	–	3.72%	3.35%	4.82%	-	4.36%	–	6.93%	4.64%
R^2^_acum_	34.84%	30.32%	27.11%	36.60%	34.15%	42.73%	18.49%	36.04%	32.54%

*ALT* altitude, *SWA* soil water availability, *PHP* photoperiod, *Pa* accumulated precipitation, *CC* clay content in the soil, *Tmax* maximum air temperature, *Tm* mean air temperature, *Tmin* minimum air temperature, *UR* relative humidity. Measurement of RH based on two meters above ground level; *and**Significant values at 5% or 1% probability; and ^ns^non-significant values; Null values (–) correspond to non-significant correlations (p > 0.05).

Regarding ALT, there was a coefficient of 0.4658 (p < 0.05), which suggests that a gradual increase of 100 m in altitude implies an average yield increase of approximately 46 kg ha^−1^. Among the cultivars, G-06 showed the greatest responsiveness to variations in altitude, with a coefficient of 1.4614 (p < 0.05) (Table 4). Regression coefficient associated with DHS was 4.7510 (p < 0.05), indicating that a 10% increase in SWA results in an increase in yield of approximately 47 kg ha^−1^. Regarding PHP, there was an average coefficient of 2.2897 (p < 0.05), which indicates that variations in photoperiod by around 2 h result in yield variations close to 5 kg ha^−1^. Among the cultivars, G-22 was the most responsive to variations in PHP, with a coefficient of 5.2780.

Regarding Pa, there is a mean coefficient of 1.6121 (p < 0.05). Taking an average accumulated rainfall of 100 mm month^−1^ as an example, there is an increase in yield by around 160 kg ha^−1^. Among the cultivars evaluated, G-20 showed the best responsiveness, with a coefficient equal to 3.4971 (p < 0.05) (Table 4). Regarding CC, there was an average coefficient of 2.2911 (p < 0.05). Among the cultivars, G-07 had the highest coefficient, at 3.1945 (p < 0.05).

Among the cultivars, G-25 showed the greatest response to variations in RH, with a coefficient of 3.5303 (p < 0.05) (Table 4). Regarding the covariates Tmax, Tm and Tmin, there was a significant general trend (p < 0.05), with coefficients equal to − 2.1475, 1.7906 and 1.3651, respectively (Table 4). It should be noted that only Tmax showed a negative coefficient, indicating that an increase in maximum temperature implies a reduction in productivity, possibly because it goes beyond the crop's adaptive limits.

**Figure 1:**
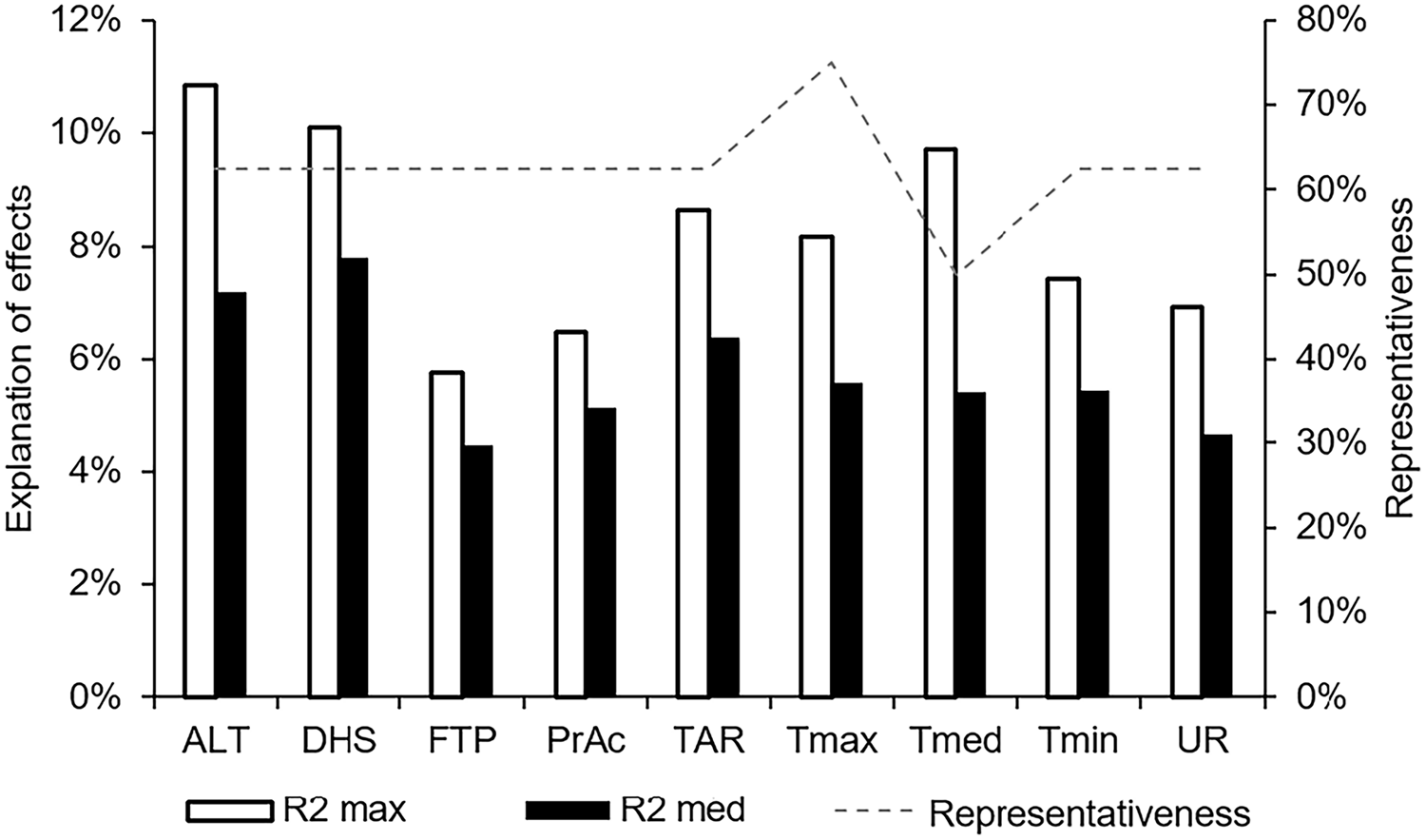
Maximum percentage contribution of significant environmental effects followed by their means, represented on the left axis. The right axis refers to the representativeness of the significant effects observed for the eight soybean genotypes evaluated in Brazil's Soybean Macroregion 4.

Figure 1 provides a graphical representation of the contribution of each variable to the phenotypic variation observed in grain yield. In this case, it is possible to see considerable differences between the mean contribution of each covariable over all cultivars in relation to the contributions at cultivar level. This finding suggests that some cultivars are more influenced by environmental factors than others.

### Geospatial distribution of grain yield

**Figure 2:**
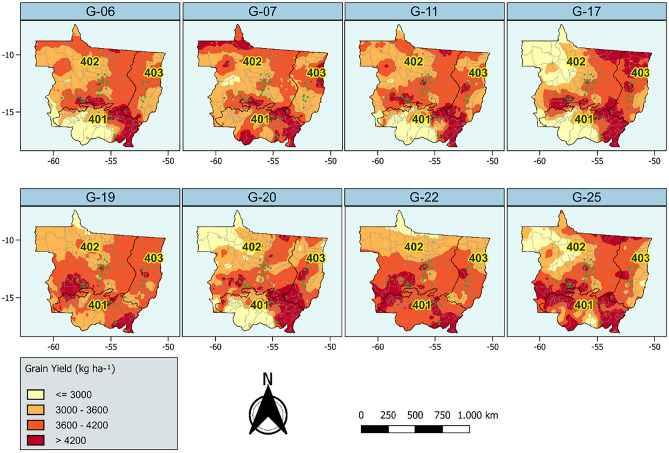
Graphical representation of the productive adaptability (kg ha^−1^) of eight soybean genotypes, evaluated in Brazil's Soybean Macroregion 4, modeled using climatic and geographical environmental variables. The adaptation of the genotypes was classified using a light (low adaptation) to dark (high adaptation) color gradient; for more details, see appendices B and C.

Figure 2 shows the adaptive geographical distribution of each cultivar. It is noteworthy that, for most cultivars, the southern region of Mato Grosso showed the highest predicted yield levels. Evidently, this is due to the higher environmental quality observed in that region. It can also be seen that the highest yields, overall, tend to be concentrated in the regions with the highest concentration of soybean cultivation. This justifies the migratory behavior of soybean cultivation in some regions, depending on their suitability for cultivation.

### Optimized recommendation of cultivars

**Figure 3:**
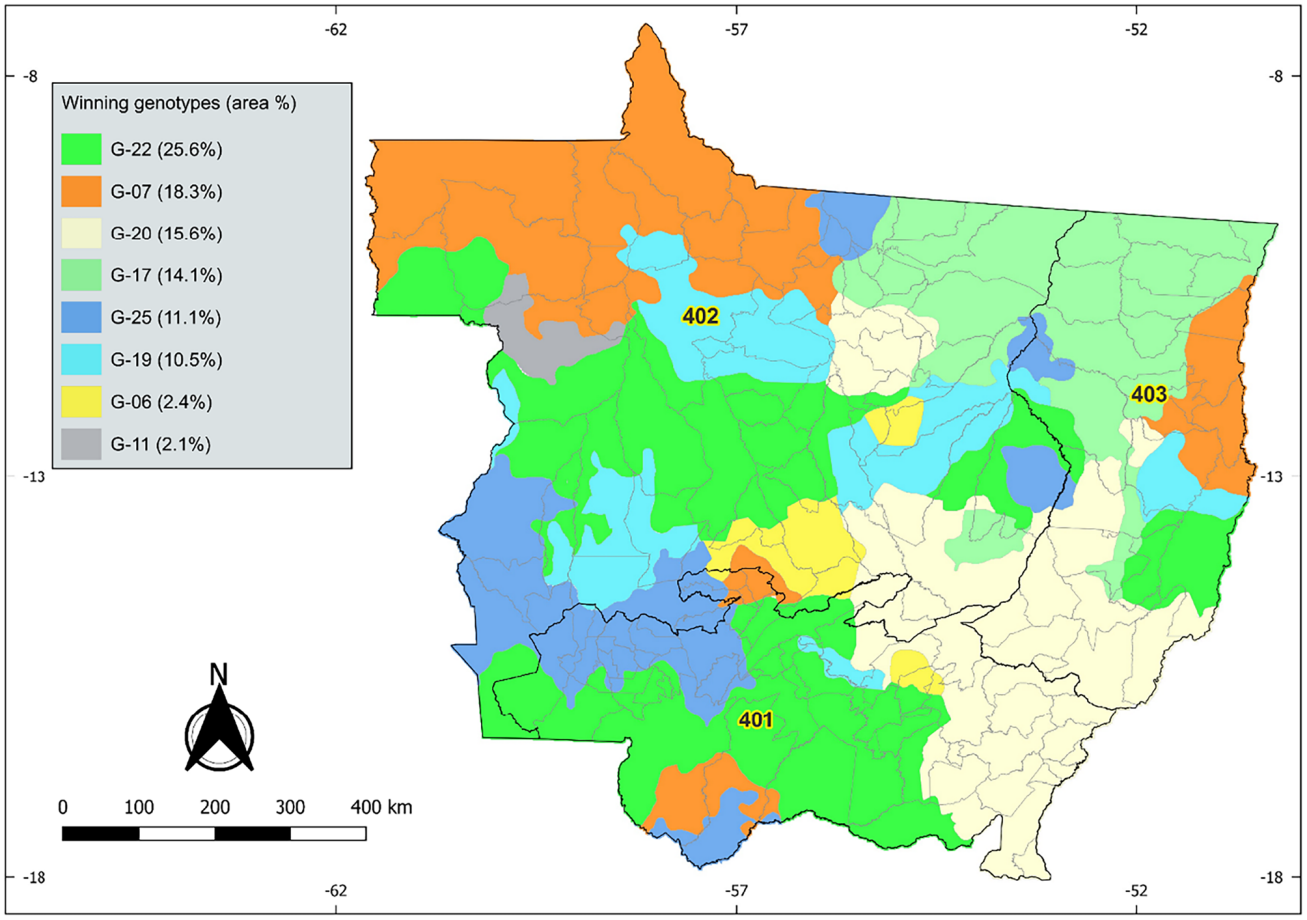
Graphical representation of the adaptation of the winning soybean genotypes in the state of Mato Grosso ("who-won-where" map). Percentage values refer to the proportion of area in which the genotype outperformed the others in the target region.

Genotypes G-19 and G-22 showed better yield adaptability, as they showed little variation in the yield adaptability classes (Fig. 3). The map shows that these two genotypes were well adapted to the three micro-regions (401, 402 and 403) with predicted mean grain yields above 3000 kg ha^−1^. Both showed a prediction higher than the mean of the eight best genotypes (4105 kg ha^−1^) for the environments Parecis, Tangará da Serra, Alto Guaporé, Alto Araguaia, Primavera do Leste, and Rondonópolis micro-regions, mainly.

Based on the predictive maps, the G-06 and G-11 genotypes showed a critical yield limit of less than 3000 kg ha^−1^ for the environments in the Center-South and Southwest mesoregions of Mato Grosso, corresponding to the Alto Guaporé, Alto Pantanal, Cuiabá and Jauru microregions, which are low production regions. On the other hand, in the Southeast mesoregion, the micro-regions of Alto Araguaia, Primavera do Leste, Rondonópolis and Tesouro, as well as part of the Parecis micro-region, located in the Northern Mato Grosso mesoregion, performed well, exceeding the average of 4200 kg ha^−1^, as shown in Fig. 3.

Regarding the northeastern mesoregion of Mato Grosso, located in macroregion 403, corresponding to the microregions of Norte Araguaia, Médio Araguaia and Canarana, genotype G-17 stood out with the best predicted production performance. In addition to these regions, this material also performed well in the microregions of Colíder, Primavera do Leste, Tesouro, Alto Araguaia and partially in the microregions of Rondonópolis, Parecis, Tangará da Serra and Paranatinga. The environments with adaptive limits for this material were the micro-regions Alto Guaporé, Alto Pantanal, Aripuanã, Jauru and partially Alta Floresta, Arinos, Cuiabá and Tangará da Serra.

**Figure 4:**
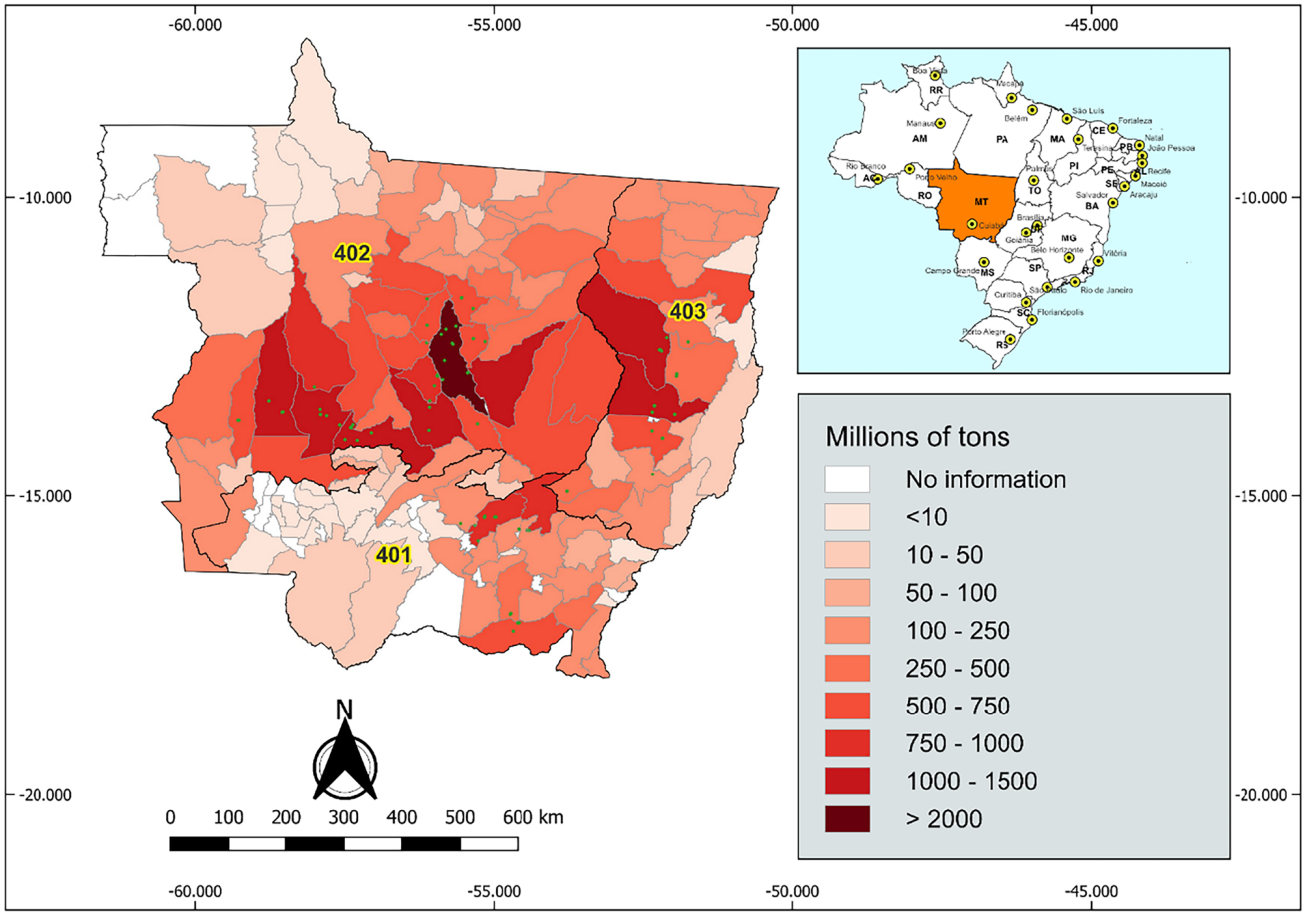
Geographical distribution of field trial sites (green dots) for the 2019/2020 to 2021/2022 harvests. The orange highlights indicate soybean production, in millions of tons, by municipality throughout the State of Mato Grosso.

Figure 4 also shows the proportions of the map covered by each cultivar, which are winning% indicators, but in a different approach than Table 2. Table 2 shows that the cultivar with the highest winning% is G-19, with 28.09%. However, when comparing with Figure 4, it can be seen that G-22 showed the highest winning % throughout Mato Grosso. It's worth noting that not the entire extension of Mato Grosso is relevant for soybean cultivation. Furthermore, there are areas on the map (Fig. 4) that are not cultivated due to edaphoclimatic limitations and, in other cases, they are earmarked for environmental conservation.

It can be seen that the G-22 genotype was the winner in 25.6% of the study area, with better adaptation in regions of high production relevance, covering the micro-regions of Parecis (Sapezal, Campos de Júlio and Comodoro); Aripuanã (Brasnorte); Arinos (Nova Maringá, São José do Rio Claro and Porto dos Gaúchos); Alto Teles Pires (Nova Mutum, Santa Rita do Trivelato, Lucas do Rio Verde, Tapurah, Ipiranga do Norte, Itanhangá and north of Sorriso); Sinop (Sinop and Vera); Paranatinga (Gaúcha do Norte); Canarana (east of Querência and Canarana) and Rondonópolis (Itiquira and Rondonópolis).

The G-20 cultivar showed the best adaptation in 15.6% of the territory of Mato Grosso, especially in the micro-regions of Parecis (Campo Novo do Parecis and Campos de Julio); Alto Teles Pires (Santa Rita do Trivelato and Nova Ubiratã); Sinop (Sinop, Cláudia and Santa Carmem); Paranatinga (Paranatinga); Canarana (Água Boa, Canarana, Ribeirão Castanheira, Santo Antônio do Leste and Novo São Joaquim); Primavera do Leste (Campo Verde and Primavera do Leste) and Alto Araguaia (Alto Garças). Possibly because it is the cultivar best suited to the regions bordering Mato Grosso and Goiás, this cultivar is adapted to Goiás.

The G-07 cultivar, despite being the most adapted in 18.3% of Mato Grosso's territory, shows the best yields in less important regions for soybean cultivation, with potential for use in the transition between Mato Grosso and Pará. The G-17 cultivar is the most adapted in 14.1% of the territory, with the highest concentration in Edaphoclimatic Region 403, mainly in the region of Canarana and other adjacent areas. It can be seen that the adaptation of this cultivar can be extrapolated to the North and Northeast regions, towards Pará and Tocantins. These adaptive behaviors highlight the opportunities for extending the use of these cultivars.

Adopting the "Who-Won-Where" approach, we can see which regions are the best adapted for each cultivar (Fig. 3). In this way, it is possible to optimize the recommendation of the best cultivars in each geographical position across the territory of Mato Grosso, aiming at maximizing grain yield. The maps for each genotype behaved differently from one another, so by visual analysis, the best adaptation zones for the genotypes can be distinguished, given the spatial variation of the environment along the geographical gradient covered by the study.

Considering the two approaches, (i) overall mean yield of the cultivars plus winning% (Table 2) and (ii) spatial analytical approach with indication of the most adapted cultivars (Fig. 3), it can be seen that using the different approaches can result in different recommendations. For example, considering approach i, the recommendation is directed towards cultivar G-19, which obtained the highest mean yield and winning of 28%. However, taking approach ii, the predominant recommendation would be for cultivar G-22. Therefore, for a more accurate cultivar recommendation, it is suggested that the spatial approach be used to complement the punctual analysis.

## Discussion

The GxE interaction suggests different adaptive capacities of the cultivars in response to environmental variations. The E effect significance indicates variations in the behavior of the cultivars, regardless of the G × E interaction. Therefore, these are environmental differences that lead to mean phenotypic changes for all the cultivars. Environmental effects (E) accounted for 31.5% of the phenotypic variation observed for grain yield. While the main genetic effects (G) accounted for 16.29% and the interaction effects (G × E) accounted for 16.61%. Thus, the ratio between G × E/G is 51%, which indicates reasonable adaptive levels of the cultivars across the environments sampled. Quantifying these values is key to understanding phenotypic adaptation and regionalizing cultivar recommendations.

The sensitivity of soybeans to photoperiod and temperature, among other factors, contributes substantially to the increase in variance associated with the GxE interaction (Table 1). This results in a greater number of cultivars needed to adequately cover a target region in order to maximize yields. Unlike other species, such as maize^13^, cotton^14^ and sugarcane^15^, which have lower G × E interaction and, consequently, greater adaptive amplitude than soybeans. Furthermore, species whose materials are used for cultivation have a higher level of heterosis or ploidy, and generally have greater adaptive capacity (homeostasis), either due to allelic variability or the number of copies of each allele^16^.

There is considerable proximity between the mean yields (Table 2), resulting from the eight high-yielding cultivars in which the most productive cultivar was G-19 (Table 3). The most productive cultivar (G-19) is usually calculated as a simple proportion, considering only the locations sampled. This analysis can be misleading, as it is assumed that a grouping of environments in a region represents all the existing environmental variation. From these perspectives, new methodologies for these findings must be proposed, with the presentation of this study.

However, considering only the predicted mean for all environments, it is not possible to visualize the distribution of this cultivar's yield across the geographical extension of Mato Grosso. It is understood that the G × E interaction is a factor that arises from eco-physiological processes, which is specific to each genotype. Knowing that gene responses vary across environments, it is possible to capture these environmental variations and associate them with these responses to find the reaction norm for each genotype.

Several traits can influence the adaptive capacity of a cultivar, especially relative maturity^17,18^. However, in this case, there was no direct association between RM and average yield (r = − 0.09^ns^), unlike what was observed by^19^. In terms of adaptability and stability, it is possible that later materials show more favorable behavior, as observed by a positive correlation between groupings of cultivars of longer maturity in relation to yields (r = 0.4168; p < 0.05), indicating that late materials tend to be predominantly more productive. This occurs because the region in which the genotypes were evaluated has predominantly warmer temperatures than in the southern region of the country (predominantly mild climate), therefore soybeans take a longer thermal time (increasing degree-days), therefore exploring maturity groups Late crops subjected to these conditions can maximize soybean grain yield^20^.

Environmental characterization is essential for understanding phenotypic adaptation. Analytical methods linked to network trials, detailed with environmental effects (environmental characterization), can support the decision-making process in order to allow predictions of productive performance along a geographical gradient^18^. This requires understanding the interference of each environmental variable on the yield of cultivars, in order to know the adaptive limits in the face of environmental variations. Bello et al.^19^ suggested the use of environmental covariates in factorial regression to explain the G × E interaction.

The behavior of soybeans can be influenced by the ALT of the crop, mainly due to the indirect relationship between this variable and the average atmospheric temperature. It is known that the higher the elevation, the lower the average temperature. Low-altitude regions tend to have predominantly higher temperatures. Physiologically, soybeans depend on an accumulation of thermal units, or degree days, for the regular progression of the phenological stages. Therefore, later cultivars tend to demand greater heat accumulation, while earlier cultivars behave differently under the same conditions. Thus, it is necessary to understand the characteristics of each environment and recommend the cultivar with the most favorable response.

Among the cultivars, G-06 showed the greatest responsiveness to variations in altitude, with a coefficient of 1.4614 (p < 0.05) (Table 4). Nascimento et al.^14^ observed significant and distinct responses between cultivars when comparing different maturity groups when grown at different altitudes.

Sufficient SWA according to cultural demand, is essential for obtaining high yields in soybeans. However, there is a high degree of variation in soil water levels over the years, due to variations in the weather and the seasons. In dryland production systems, sowing is usually done at times that coincide with sufficient water availability. Therefore, the sowing date for soybeans should result in a synchronization between the variations in the levels of SWA and the crop's water demand, considering cultivars adapted to a given target region. Sentelhas et al.^18^ observed, mainly in Southern Brazil, a reduction of more than 1600 kg ha^−1^ in crop yield due to SWA. Additionally^20^, when estimating variations in productivity due to water stress, found losses around 46%. In our study, over the years analyzed, we did not find such extreme conditions compatible with losses of this magnitude.

FTP is the most important environmental factor for soybean adaptation, as it can substantially affect the plant's growth and development dynamics. This behavior stems from the photosensitivity of soybeans, which results in the induction of flowering under short-day conditions. However, the soybean crop has a time interval during the first phenological stages in which it is photoneutral, known as the juvenile period. Thus, for a cultivar to be adapted to a region, showing reasonable size and yields, there must be an adjustment between the length of the juvenile period and the occurrence of the critical photoperiod in the growing region. The crop's wide adaptability to different latitudes is due to genetic variation associated with genes controlling the juvenile period and impacting, together with others, flowering and maturation^21^. Gupta et al.^21^ pointed to the discovery of the variability and genetic control of the juvenile period as the most crucial aspect for soybean progress in Brazil, which made it possible to develop cultivars adapted to low latitudes.

Obviously, Pa has a strong relationship with variations in SWA, Tm, RH, among other factors. Thus, precipitation results in complex effects between different environmental factors. Water stress triggers several physiological processes, such as inhibition of photosynthesis, transpiration and stomatal conductance, which affect growth, development and consequently crop yields^23^. de Souza Nóia and Sentelhas^22^ found that water deficit is the main cause of the soybean yield gap in Brazil. The study revealed that would be possible to increase the average yield by 927.5 kg ha^−1^ under conditions free from water stress in Mato Grosso.

CC mainly impacts the dynamics of nutrients and water flow in the soil, interfering with fertility levels, thermal variation, and soil evaporation. Therefore, soybean adapts better to medium-textured soils, preferably soils with a CC between 30 and 35%^23^. More demanding cultivars, responsive in environments with a higher technological level, tend to show better results at these clay content rates in the soil. Assuming that the CC in the main arable regions of Mato Grosso is between 15 and 60%, there are variations between 45 and 180 kg ha^−1^. Clearly, the effects of CC are more significant under extreme conditions of SWA, but interaction effects between covariates were not addressed in this study. In research carried out to assess the impacts of climate change on the development of soybean crops, the authors performed simulations of water and thermal stress and observed that under lower rainfall conditions, the region's soil, characterized by its high clay content, stored enough water for the regular development of soybeans and, consequently, the maintenance of yield levels^24^.

The models predicting crop responses to rising temperatures suggest that a future warming climate should be detrimental to yields in most of the regions relevant to soybean cropping^25^. However, there are uncertainties about these predictions based on spatial and temporal variations in the increase in temperatures over the crop seasons 34. In this context, genetic breeding aimed at developing cultivars that are tolerant to abiotic stresses, especially excess heat, is one of the key tools for making farming feasible in global warming scenarios^26,27^.

Although RH is closely related to Pa and SWA, it has a major impact on phytosanitary aspects of the crop. In many cases, RH modulates pathogen-host relations, providing or not a favorable environment for the occurrence of diseases. However, there was a positive mean coefficient (p < 0.05) of 1.3559. Among the cultivars, G-25 showed the greatest response to variations in RH, with a coefficient of 3.5303 (p < 0.05) (Table 4). Assuming that the mean RH in the crop season is equal to 80%, with decreases to levels close to 40% during periods of drought, this covariate can result, on average, in variations of approximately 54.4 to 108.4 kg ha^−1^. In this case, it is possible that the effects of this covariate are complementary to those associated with SWA and Pa. Zhao et al.^28^ observed that RH above 80% is favorable for soybean yields, but under these environmental conditions, there was also a favorable environment for pathogen incidence.

The proportion of the GxE interaction explained by the covariates is 32.54% (Table 5). We therefore assume that other covariates, not considered here, are associated with the complementary proportion of the G × E interaction, as well as possible complex interactions between them. Also, in the multiple linear regression procedure, it is assumed that the effects are linear and additive. Thus, any different behavior is not capitalized on when adjusting the model. Furthermore, there are other variables associated with soybean management that differ at a local level and which have not been addressed in the methodology of this study. Becker and Leon^29^ found that, usually, just a minor proportion of the G × E interaction can be attributed to known environmental factors, with the greater proportion remaining unclear in multi-site yield trial analyses. Verhulst et al. and Ortiz et al.^30,31^ observed superior results, reaching proportions of 90.20% and 91.16% of the G × E interaction explained by environmental covariates in the wheat and tomato crops, respectively.

**Table 5:** Relevant environmental covariables for soybean adaptation used in the study.

Covariable	Abbreviation	Data source	Unit
Accumulated rainfall	Pa	CHIPS2 (5.0 km)	mm
Soil water availability	SWA	ERA5 (30.0 km)	Decimal
Photoperiod	PHP	ERA5 (30.0 km)	Minutes
Maximum temperature	Tmax	ERA5 (30.0 km)	°C
Mean temperature	Tm	ERA5 (30.0 km)	°C
Minimum temperature	Tmin	ERA5 (30.0 km)	°C
Relative humidity^1^	RH	ERA5 (30.0 km)	%
Clay content^2^	CC	SOILGRIDS (0.25 km)	%
Altitude	ALT	NASA	Meters

^1^Observation at two meters above the ground; ^2^Layer 0–15 cm deep.

Due to the G × E interaction, it is common for some cultivars to perform better than others in certain locations. However, methods for analyzing multi-location yield trials generally allow for specific interpretations at the site level or at the environmental grouping level. By adopting geospatial approaches to gain a better understanding of yield variation across geographic expanses in production regions, there is greater visibility into the behavior of cultivars. Furthermore, with the help of environmental covariates, it is also possible to predict the adaptive potential of soybean cultivars in other growing regions and to guide the extension of their use.

The G-19 cultivar showed more restricted adaptation, but in areas of high relevance for soybean production, being the most adapted in considerable proportions of the Parecis and Alto Teles Pires regions. It should be noted that this cultivar showed the best mean performance in the yield trials when analyzed individually (Table 2).

Thus, it can be seen that the environmental covariates adopted in this study explain around 35% of the G × E interaction. Thus, this approach can provide a better understanding of the adaptation of cultivars and contribute to more assertive recommendations. Geographical adaptability can complement strategies for selecting and recommending cultivars by demonstrating regions of greater adaptation for each cultivar, as well as indicating possible adaptive tendencies to support extensions of use.

The results of this research highlight that the G-19 and G-22 genotypes exhibited remarkable productive adaptability, showing consistent performance in several microregions, especially in 401, 402 and 403. In contrast, the G-06 and G-11 genotypes revealed productivity restrictions, particularly in the Center-South and Southwest mesoregions of Mato Grosso, where production is historically lower. However, they demonstrated satisfactory performance in areas in the North of Mato Grosso. Cultivar G-07, although it has shown comprehensive adaptation in a large part of the Mato Grosso territory, has demonstrated to be more productive in regions less relevant for soybean cultivation, suggesting a potential application in transition areas between Mato Grosso and Pará. By In turn, cultivar G-17 exhibited significant adaptation, mainly in the Edaphoclimatic Region 403, indicating possibilities for use in the North and Northeast regions, towards Pará and Tocantins. Finally, the G-19 cultivar, despite its more restricted adaptation, revealed superior performance in strategic areas for soybean production, emphasizing its relevance in specific cultivation scenarios. These findings highlight the importance of careful selection of adapted genotypes to optimize the productivity and efficiency of soybean in different regions of Mato Grosso. These findings reinforce the importance of the careful selection of genotypes adapted to the specific conditions of each region, aiming to optimize the grain yield and efficiency of soybean crops in Mato Grosso.

Detailed analysis of the predictive maps revealed that the G-22 genotype was particularly successful in an extensive area, covering microregions of great productive relevance. Similarly, the G-20 genotype stood out in a significant portion of the Mato Grosso territory, especially in microregions such as Parecis, Alto Teles Pires, Sinop, Paranatinga, Canarana, Primavera do Leste and Alto Araguaia, demonstrating its adaptation and yield potential. Grain yield maximization maps, based on cultivar use zoning, can make a substantial contribution to improvement strategies through a better understanding of cultivar competitiveness at a geospatial level. Furthermore, it helps direct efforts to develop new cultivars adapted to challenging regions.

## Methods

### Characterization of the region and study area

The data used in this study comes from yield trials of the Syngenta Seeds soybean breeding program, conducted in 28 locations in the State of Mato Grosso, in relevant soybean production regions, covering the Edaphoclimatic Region (REC) 401, 402 and 403 (Fig. 4). The trials were conducted over three years, during the 2019/2020, 2020/2021 and 2021/2022 crop seasons. The region targeted by the study stands out for its large territorial extension and its inclusion in three biomes: Amazon, Cerrado and Pantanal, reflecting high heterogeneity due to different edaphoclimatic and biotic conditions present in the environments of this region. It has two well-defined seasons: rainy and dry, with the rainy season running from October to April and the dry season from May to September. The average annual rainfall in the state ranges from 1200 to 2200 mm, with the highest figures in the north of the state.

### Experimental network and phenotypic data

Trials were carried out in a randomized complete block design with three replications. Thirty-two soybean genotypes (commercial and pre-commercial) representing different maturity groups (7.5–8.5) were evaluated in each trial. Each replication consisted of four 5 m long rows spaced 0.5 m apart. Useful plot area was 10 m^2^ and grain yield was determined by harvesting the entire plot. Grain yield was then estimated (kg ha^−1^) at 13% moisture content.

Trials were managed according to the technical recommendations for soybean cultivation in Brazil. In all cases, sowing was carried out at the appropriate dates for each region, following the recommendations of the Agricultural Climate Risk Zoning (ZARC) for the State of Mato Grosso.

### Environmental covariables

Environmental covariables were obtained from the Meteoblue system after prior selection of covariables based on the redundancy level, analyzed pair by pair, and correlation analysis with yield. None of the regression models generated with covariates showed severe multicollinearity. Thus, the geographical and environmental covariables considered in this approach were: accumulated precipitation (Pa), minimum temperature (Tmin), mean temperature (Tm), maximum temperature (Tmax), photoperiod (PHP), relative humidity (RH), clay content (CC), soil water availability (SWA) and altitude (ALT) (Table 5).

For obtaining the covariables, a polygon (vector file) was first built with a 10 km × 10 km grid, totaling 15,113 points across the State of Mato Grosso. Subsequently, each variable was interpolated using the IDW method (*Inverse Distance Weightening*). It should be noted that the interval from October 1 to March 15 was used to delimit the useful soybean season over the three years. Therefore, the covariables adequately represent the environmental conditions occurring in the harvests.

Regarding the CC variable, a 0–15 cm depth layer was considered. For this purpose, static behavior was assumed for this variable, given that this information shows variations over the three years considered in the study. As for altitude, we used the "digital elevation model"—SRTM 41, with a similar resolution to the other variables.

### Statistical analyses

Initially, the Linear Mixed Model (LMM) (1) was applied, followed by the respective deviance analysis adopting a 5% significance level. Subsequently, the fitted mean yields for each cultivar were obtained, as well as the effects associated with the sources of variation genotype and genotype x environment interaction. For this purpose, the *lme4* package of the R software^32^ was used for the model fittting.

1 $$ Y_{ijk} = \, u \, + \, a_{i} + \, b_{j/i} + \, g_{k} + \, ga_{ik} + \, e_{ijk} $$

wherein: *Y*_*ijk*_: yield observed in each plot; *u*: model intercept; *a*_*i*_: random environment effect; *b*_*j/i*_: random and hierarchical effect of block within environment; *g*_*k*_: random genotype effect; *ga*_*lk*_: random genotype × environment effect; *e*_*ijk*_: experimental error, assumed to be *e*_*ijk*_ ~ N(0, σ^2^).

After adjusting the means of each cultivar at a local level, the Regression–Kriging method^33^ was applied or the spatialization of the yield variable using environmental covariates, according to the equation (2):

2 $$\widehat{z}{(s}_{0})=\widehat{m}{(s}_{0})+\widehat{e}{(s}_{0})$$

$$={\sum }_{k=0}^{p}{\widehat{\beta }}_{k} .{\widehat{q}}_{k}{(s}_{0})+ {\sum }_{i=1}^{n}{\widehat{\uplambda }}_{i}. e{(s}_{i})$$

wherein: $\widehat{z}{(s}_{0})$: prediction of the regionalized variable in an unsampled position ${s}_{0}$; $\widehat{m}{(s}_{0})$: partial fitting, by regression, in a given neighborhood (*trend*); $\widehat{e}{(s}_{0})$: residual interpolated by kriging; ${\widehat{\beta }}_{k}$: generalized least square estimates ($k=0, 1, 2, \dots p$) of the partial regression coefficients associated with the p selected predictor variables; ${\widehat{q}}_{k}{(s}_{0})$: are the values assumed by the predictor variables (environmental covariables) in position ${s}_{0}$, with ${\widehat{q}}_{0}{(s}_{0})=1$; ${\widehat{\uplambda }}_{i}$: are the weights derived from the spatial structure of residuals; $e{(s}_{i})$: is the residual of the multiple linear regression at a sampled position ${s}_{i}$.

Firstly, the *ga*_*ik*_ interaction was spatialized and the values obtained were then added to the main effects of genotypes and intercept. In this way, the predictor *Y*_*ij*_ = *u* + *g*_*i*_ + *ga*_*ij*_ was used for obtaining means at each geographical coordinate. This procedure was carried out using the softwares SAGA^34^ and R^32^, exploring the *rsaga* package^35^, and the maps were subsequently drawn up using Quantum GIS software.

Grain yield was spatialized only for the eight most productive cultivars, based on the overall mean in all the locations sampled. Subsequently, a layer representing the adaptive delimitations of each cultivar was obtained based on the maximum yield values. In this way, the optimized map for recommending cultivars was drawn up.

## Data Availability

The datasets used and/or analysed during the current study available from the corresponding author on reasonable request.
